# Differences in the Values and Related Factors of Eating a Balanced Meal among the Younger Generation in Japan

**DOI:** 10.3390/nu16121855

**Published:** 2024-06-13

**Authors:** Fumi Hayashi, Yukari Takemi

**Affiliations:** Faculty of Nutrition, Kagawa Nutrition University, 3-9-21 Chiyoda, Saitama 350-0288, Japan; takemi@eiyo.ac.jp

**Keywords:** values, motivations, dietary behaviors, healthy eating, young adults, Japan

## Abstract

This study examined young Japanese adults’ values regarding the consumption of balanced meals consisting of staples, main dishes, and side dishes and how these values relate to demographics, socioeconomic status, nutrition knowledge, attitudes, skills, behaviors, and diet-related quality of life. Data were obtained from the “Survey on Dietary Habits of the Younger Generation” (2000 responses, ages 18–39), of which 1888 valid responses were analyzed. The principal component analysis identified three value patterns: PC1—valuable yet burdensome; PC2—environment-reliant, weak initiative; and PC3—low value due to hassle. Both PC1 and PC3 were associated with prioritizing prices in food choices and knowledge of a balanced meal. However, PC1 participants valued balanced meals and possessed meal preparation skills, whereas PC3 participants valued balanced meals less and had negative attitudes toward eating them. PC1 was positively associated with the frequency of eating balanced meals while PC3 was negatively associated. PC2 individuals had positive attitudes toward eating balanced meals but were less concerned about nutritional balance when choosing foods themselves. This study highlights the importance of adopting an approach that aligns with the value patterns of the target population.

## 1. Introduction

Healthy eating is vital in preventing non-communicable diseases. Poor diet, overweight, and obesity are major risk factors for diabetes, cardiovascular disease, and cancer [1]. Adherence to healthy eating patterns, such as the Mediterranean Diet, Healthy Eating Index, or Dietary Approaches to Stop Hypertension, is associated with lower mortality risks among adults [2,3]. Adherence to the Japanese Food Guide Spinning Top, which recommends having a balanced meal consisting of staples, main dishes, and side dishes, is also known to lower the total and cerebrovascular mortality risks [4] and frailty risks [5] among middle-aged and older Japanese adults.

Research has identified that young adults globally have poor diet quality compared to other adults [6,7,8,9], with high consumption of ultra-processed foods [6] and low intake of fruits and vegetables [7] and whole grains [8]. Furthermore, early adulthood is a period associated with poor eating habits and rapid weight gain. This is because early adulthood is a transitional period that includes environmental, social, and lifestyle changes that may affect dietary changes [10]. Leaving home and education are associated with negative dietary changes. Therefore, further research is needed to identify and understand specific segments within the young adult age group, which is particularly prone to dietary changes, to develop tailored strategies for promoting balanced diets. Encouraging a healthy diet from an early age is essential for overall well-being [11]. Individual lifestyle patterns that develop during this transition often persist later in life. Although this age group is prone to dietary changes due to life events such as marriage and parenting, which may have health implications [12], young people’s health has often been neglected in global public health because this age group is perceived as healthy and is difficult to reach.

To improve dietary intake, it is necessary to explore the determinants of dietary behavior and their influence on diet quality. Diet changes over time, and food preparation behaviors persist. Involvement in food preparation during the early stages of adulthood has been linked to healthier dietary intake in the mid-to-late twenties [13]. However, individuals’ dietary behaviors are influenced by various factors, including intrapersonal factors (e.g., age, sex, education, lifestyle, motivation, knowledge, skills, and appetite), interpersonal factors (e.g., family support, social occasion, culture or traditions, and dietary patterns of friends or family), and environmental factors (e.g., food cost, food accessibility and availability, and media) [14,15,16]. Financial concerns are frequently reported as barriers to healthy eating [14]. Specifically, for youths, social media use is an important determinant of dietary behavior. A systematic review indicated that young adults seemed receptive to healthy eating and recipe tips through social media [17]. Encouraging consumer demand for healthy foods and diets through nutrition education and developing healthy food environments, including social media, are important.

Eating meals consisting of staples, main dishes, and side dishes at least twice a day is associated with better nutrient and food group intake and a higher likelihood of meeting the Dietary Reference Intakes for the Japanese [18]. In Japan, as part of the national health promotion initiatives, consuming such meals twice or more daily, almost daily, is an important objective. However, <40% of the adult population is engaged in this practice, and the figure is even lower among the younger population aged 20–39 years, falling below 30% [19]. To achieve this goal, it is crucial to understand the factors hindering desirable dietary behaviors and consider specific measures accordingly. While many previous studies have reported on dietary behaviors, attitudes, and socioeconomic factors related to the frequency of consuming meals with staples, main dishes, and side dishes [20,21,22,23,24,25,26,27,28,29,30,31,32,33], these studies have mostly focused only on behavioral aspects. Therefore, evidence of the differences underlying people’s values behind such behaviors is limited.

Factors reported to be related to the frequency of eating a combination of staples, main dishes, and side dishes include demographics [30,33], financial resources [20,25,28], education [28], health awareness [23,31,32], knowledge [27,32], cooking frequency [22,31] preference [27], dietary behaviors including skipping meals [30,33], and eating out [24,30]. However, no studies have evaluated the values toward eating a balanced meal and how such values are associated with individual factors. Therefore, clarifying the core components of the specific patterns of the younger generation’s values toward eating a balanced meal and its related factors could be a valuable resource for considering an approach that matches the value patterns of the target population. Therefore, this study aimed to elucidate the core components and patterns of the younger generation’s perceived value of eating a balanced meal. In addition, this study examined the factors associated with the identified value patterns, such as sociodemographic factors, food knowledge, attitudes, skills, behaviors, and diet-related quality of life.

## 2. Materials and Methods

### 2.1. Survey Methodology and Participants

We reanalyzed the data from the “Survey on Dietary Habits of Younger Generations” conducted by the Ministry of Agriculture, Forestry, and Fisheries [34]. This cross-sectional survey included 2000 responses from individuals aged 18–39 years living nationwide. The survey was conducted online in November 2019 using monitors from Cross Marketing Inc. (Tokyo, Japan). Cross Marketing Inc., a professional marketing research company, has one of the largest active registered panels in Japan, with over 5 million people, including panels registered with partner companies [35]. The survey had a target sample size of 2000 individuals (1000 males and 1000 females). Allocation by sex, age, and area of residence was determined according to the results of national census data [34]. Students were excluded because of their different lifestyles. In addition, those who had received special nutrition and culinary arts training were excluded to avoid response bias.

Before analysis, permission for data use was obtained from the government. After excluding incomplete responses, 1888 responses were analyzed.

### 2.2. Measures

We used data on sociodemographic and anthropometric variables, perceived values of eating a balanced meal, dietary knowledge, attitudes, skills, behaviors, and diet-related quality of life.

#### 2.2.1. Sociodemographic and Anthropometric Variables

The sociodemographic variables collected included demographics (age, sex, living status, marital status, and living with children) and socioeconomic characteristics (occupation, education, and household economic status). Anthropometric characteristics included body mass index (BMI), calculated based on the self-reported height and weight of the participants.

#### 2.2.2. Perceived Values Regarding Eating a Balanced Meal

A 12-item questionnaire was used to examine the participants’ perceived values regarding eating a balanced meal (a combination of staples, main dishes, and side dishes), as shown in Table 1. The participants rated the items on a 7-point Likert scale (1, not applicable at all; 7, very applicable). Participants were asked, “Please indicate the extent to which the following statements apply to your opinion of eating a combination of staples, main dishes, and side dishes, using a rating of 1 to 7 points”. Cronbach’s alpha for the 12 items was 0.900.

#### 2.2.3. Dietary Knowledge, Attitudes, Motivations for Food Choices, and Skills

Dietary knowledge regarding the consumption of a combination of staples, main dishes, and side dishes was assessed using a single item. Participants were asked, “Do you know what a “meal consisting of a combination of staples, main dishes and side dishes” is?”. Subsequently, they selected one of three answers (1: I know both the term and its meaning; 2: I do not know the meaning, but I have heard the term; and 3: I do not know the term or its meaning).

Regarding dietary attitudes, we assessed two items. First, we assessed the participants’ attitudes toward practicing healthy eating habits. Participants were asked, “Are you committed to practicing a healthy diet regularly?”. They rated their commitment on a 4-point Likert scale (1, not at all; 4, always). Second, participants were asked about eating a combination of staples, main dishes, and side dishes daily: “Please indicate how much of the following applies to your daily dietary habits by rating them from 1 to 7”. They rated the items on a 7-point Likert scale (1, not applicable at all; 7, very applicable). The following 10 items were used to assess participants’ motivations for food choices: nutritional balance, energy (calories), taste, price, seasonality, safety, appearance, word-of-mouth, popularity, and image of the manufacturer or store. Participants were asked ahead of each item, “Please indicate how much of the following applies to what you consider important when cooking or choosing meals in your daily life”. They rated the items on a 7-point Likert scale (1, not applicable at all; 7, very applicable).

Dietary skills were determined based on the following five items: considering the balance of nutrition and taste when planning a menu; knowing what ingredients and utensils are needed for preparing meals; reading nutrition labels and making food selections; ability to prepare most dishes; and ability to prepare meals in a short time, even when busy. Participants were asked ahead of each item, “Please rate the extent to which the following applies to your meal preparation knowledge and skills”. They rated the items on a 7-point Likert scale (1, not applicable at all; 7, very applicable).

#### 2.2.4. Dietary Behavior and Diet-Related Quality of Life

Regarding the dietary behavior of eating a combination of staples, main dishes, and side dishes, participants were asked, “In your daily life, how many days of the week do you eat a combination of staples, main dishes, and side dishes at least twice a day?”. They then selected one of the following four answers: (1) almost never, (2) 2 or 3 days a week, (3) 4 to 5 days a week, or (4) almost every day.

Diet-related quality of life was assessed using the subjective diet-related quality of life (SDQOL) measure, a validated four-item scale [36]. The SDQOL is composed of four items (“I enjoy mealtimes”, “I am eager for mealtimes to come”, “My meals are eaten in a positive atmosphere”, and “I am satisfied with my daily diet”). Responses were provided on a 5-point Likert scale (1, disagree; 5, agree). Cronbach’s alpha for the 12 items was 0.829.

### 2.3. Ethics Approval

The survey was administered online by Cross Marketing Inc. (Tokyo, Japan). All participants were informed that the questionnaire was given for research purposes and that their participation was completely voluntary. Because the data were collected anonymously using an online questionnaire without including personal data, written informed consent was not required. The study protocol was approved by the Ethics Committee of Kagawa Nutrition University (Saitama, Japan; approval number: 291; approval date: 17 May 2023).

### 2.4. Statistical Analysis

Using principal component analysis, we identified the value patterns of consuming a balanced meal from the 12 surveyed items. Multiple regression analysis was used to explore independent factors associated with these patterns.

The patterns of the participants’ perceptions regarding the consumption of staples, main dishes, and side dishes were analyzed as follows. Principal component analysis (PCA) with varimax rotation, including 12 items, was conducted to facilitate the interpretation of each component. The varimax rotation technique is a type of orthogonal rotation technique that results in uncorrelated components to ease the interpretation underlying the phenomenon being captured by each component [37]. Therefore, in our analyses, we used varimax rotation. Three principal components with eigenvalues > 1 were identified, and the cumulative contribution ratio was 75.1%. To identify a component as meaningful, the cumulative variance-extracted components must encompass at least 70% of the dataset’s variance [38]. Considering up to the third principal component, the principal component scores were calculated to have a mean of 0 and a variance of 1. The higher the principal component score, the higher the loadings of each of the items that make up the pattern; thus, it is an indicator of the characteristics of the composition of the pattern [38]. Following this, items displaying factor loadings beyond ±0.4 were scrutinized as having significant interpretive loadings [38]. Based on their characteristics, type names were assigned to each component. Sample validity was verified using the Kaiser–Meyer–Olkin measure of sampling adequacy (KMO measure), yielding a KMO measure of 0.879, confirming its validity.

Simple and multiple linear regression models with stepwise methods were used to describe the characteristics of each principal component pattern. The component scores were used as the dependent variables. The independent variables were sociodemographic characteristics, dietary knowledge, attitudes, motivations for food choices, skills, behaviors, and diet-related quality of life.

All analyses were performed using the IBM SPSS Statistics software version 27.0 (IBM Corp, Armonk, NY, USA).

## 3. Results

### 3.1. Participant Profile

Table 1 displays the means and standard deviations of the participants’ demographic and anthropometric variables. Among the 1888 participants, 50.8% were male, with an average age of 29.7 years (SD = 6.2). Regarding living status, 27.3% of the participants lived alone. Approximately two of three participants were single (65.1%) and did not have children (75.1%). According to educational attainment, the predominant form of education was higher education, encompassing degrees beyond the college or university level. Most participants experienced varying degrees of financial constraint, with only 9.7% reporting financial security and worry-free lifestyles. Most participants were within the normal range (BMI, 18.5–24.9); however, 19.3% were underweight (BMI < 18.5) and 13.6% were obese (25.0 ≤ BMI).

Table 2 shows participants’ profiles regarding dietary knowledge, attitudes, skills, behaviors, and diet-related quality of life. At the level of knowledge regarding eating a combination of staples, main dishes, and side dishes, nearly half of the participants (46.3%) demonstrated an understanding of both the terminology and its meaning; however, more than half of the participants lacked an understanding of the meaning (53.6%). Among the food choice motives, “taste” scored the highest (4.9 ± 1.5), followed by “price” (4.8 ± 1.5). However, a very small proportion of participants (7.5%) always prioritized the practice of healthy eating habits. Regarding eating behaviors, approximately one of four participants (24.8%) reported consuming a combination of staples, main dishes, and side dishes twice or more daily, almost every day. Alternatively, a similar percentage of respondents (26.3%) indicated that they rarely do so.

### 3.2. Principal Component Analysis of Perceptions Regarding Eating a Balanced Meal

We analyzed 12 measures of the participants’ perceptions regarding eating a combination of staples, main dishes, and side dishes using principal component analysis with varimax rotation. The three components had eigenvalues > 1.0, accounting for 75.1% of the total item variance. The Kaiser–Meyer–Olkin measure of sampling adequacy was high (KMO = 0.879).

As shown in Table 3, the first component was labeled “Valuable yet burdensome” (PC1: Eigenvalue = 5.85, 48.7% item variance). PC1 had high positive loadings (0.828–0.878) for three components that considered a balanced meal healthy, nutritious, and fulfilling for participants. In addition, it had moderately positive loadings (0.429–0.733) regarding positive attitudes toward eating if meals were prepared or if someone was eating them. However, it also had moderately positive loadings (0.483–0.544) for feeling the burden of preparation and adoption. The second component was labeled ‘environment-reliant, weak initiative’ (PC2: Eigenvalue = 1.90, 15.8% item variance). This component showed moderate to high positive loadings (0.662–0.872) on items such as meals participants would use if they were available or easily purchased. The third component was labeled “Low value due to hassle” (PC3: Eigenvalue = 1.26, 10.5% item variance). PC3 had high positive loadings (0.687–0.791) for three components regarding burden: preparing a balanced meal wastes time, is troublesome, and is costly. Although the factor loadings were small, there were negative loadings for eating a balanced meal to build relationships with others or eating when others eat.

### 3.3. Factors Associated with Participants’ Perception Patterns Regarding Eating a Balanced Meal

Table 4 shows the linear regression coefficients from simple and multiple analyses, examining factors associated with participants’ perception patterns regarding eating a balanced meal. The multiple linear regression analysis reveals that factors associated with the PC1 pattern include being female (β = 0.040, *p* < 0.05), being married (β = 0.052, *p* < 0.01), having sufficient knowledge (β = 0.091, *p* < 0.001), having a positive attitude (β = 0.090, *p* < 0.001), and possessing skills for preparing meals (β = 0.114, *p* < 0.001). Additionally, food choice motives such as taste (β = 0.259, *p* < 0.001) and price (β = 0.199, *p* < 0.001) were positively associated with this pattern, while participants were less likely to consider popularity (β = −0.110, *p* < 0.001) and word-of-mouth (β = −0.079, *p* < 0.001) when choosing foods. The adjusted R^2^ was 0.411 in the multiple model.

Analysis of the PC2 pattern revealed negative associations with sociodemographic variables and food-choice motives. Individuals who had higher component scores for this pattern were more likely to be young (β = −0.060, *p* < 0.01), male (β = −0.070, *p* < 0.001), living alone (β = −0.077, *p* < 0.001), and have lower motives for nutritional balance (β = −0.116, *p* < 0.001) and taste (β = −0.056, *p* < 0.05) when selecting foods. On the other hand, individuals in this pattern showed higher scores for attitudes toward eating a balanced meal (β = 0.105, *p* < 0.001) and higher concerns for selecting foods with good manufacturer or store brand image (β = 0.240, *p* < 0.001) or popularity (β = 0.142, *p* < 0.001). The adjusted R^2^ was 0.268 for the multiple models.

Lastly, the linear regression analysis for the PC3 pattern demonstrated a positive association with age (β = 0.052, *p* < 0.05) and knowledge (β = 0.095, *p* < 0.001) but a negative association with household economic status (β = −0.065, *p* < 0.01) and attitudes toward eating a balanced meal (β = −0.142, *p* < 0.001). In terms of food choice motives, this pattern was more likely to be concerned with price (β = 0.298, *p* < 0.001) and taste (β = 0.103, *p* < 0.001) but less concerned with seasonality (β = −0.168, *p* < 0.001). The adjusted R^2^ was 0.195 for multiple models.

In terms of behavior, a higher frequency of eating a balanced meal was observed in the PC1 pattern (β = 0.043, *p* < 0.05), while a lower frequency was observed in the PC3 pattern (β = −0.122, *p* < 0.001). Furthermore, a higher SDQOL score was associated with PC1, while a lower score was observed in PC3. No significant association was observed with the PC2 pattern.

## 4. Discussion

### 4.1. Value Patterns and Related Factors

This study identified three patterns and components of perceived value among young adults regarding the consumption of a balanced meal consisting of staples, main dishes, and side dishes more than twice daily. Furthermore, we identified the independent factors associated with each pattern. Different characteristics were observed for each pattern. Individuals with high component scores in the PC1 pattern believed that eating a balanced meal was important for their health and wanted to incorporate it; however, they also felt a sense of burden. Despite facing time and financial constraints, individuals with high PC1 scores had a higher frequency of consuming a balanced meal in practice because they possessed the necessary knowledge, skills, and attitudes for meal planning. Those with higher PC2 component scores were more motivated to eat a balanced meal. However, nutritional balance and taste were not prioritized in their actual food choices, and there was no significant association with meal preparation skills. Consequently, there was no association with the frequency of eating balanced meals. Finally, individuals with higher PC3 scores who were less likely to value a balanced meal because of the hassle of cost, effort, and time investment were negatively associated with the practice of a balanced meal. In addition, those who scored higher in this pattern were found to lack financial comfort and to be more concerned with taste, price, and brand image when making food choices. To our knowledge, this is the first study to identify the core components and patterns of individuals’ values regarding eating a balanced meal and how they relate to sociodemographic factors, dietary knowledge, attitudes, skills, behaviors, and SDQOL.

Positive values for eating a combination of staples, main dishes, and side dishes were predominantly observed in participants who followed the PC1 pattern. The participants were more likely to be female or married. They were also more concerned about eating a balanced meal daily and perceive such a lifestyle as more applicable. Generally, healthier food behaviors are associated with older age, higher education, and female sex [39]. Additionally, the participants who followed PC1 and PC2 perceived several benefits of eating a balanced meal in a social context. For example, it facilitates communication with family members and increases their willingness to eat if others are eating. However, participants in PC2 were more environmentally dependent and did not value eating a balanced meal as much as those in PC1, and food-choice motives were negatively associated with nutritional balance. Therefore, no significant association was observed between PC2 and eating balanced meals. Furthermore, for PC3, the value of a balanced meal within the social context found in PC1 and PC2 was negative, indicating a negative association. Our findings suggest that different value patterns may influence actual behavior. Therefore, we explore the potential factors associated with these value differences and consider their implications for promotion strategies.

Price was an independent predictor for both PC1 and PC3 and was positively associated with both patterns. For PC3, price showed the largest regression coefficient among the independent predictors. One possible reason for the differences in actual eating behavior is the existence of economic constraints. Among the three patterns, household economic status was an independent predictor only for PC3, indicating that it was associated with greater constraints. A previous study targeting younger adults aged 20–39 years reported that, regardless of educational attainment, a poor subjective financial status increases the risk of unhealthy dietary habits, including low frequency of eating a balanced meal [28]. The tendency to prioritize price as a motivation for food selection was demonstrated in PC1 and PC3, but it was more strongly associated with PC3. It has been reported that individuals with lower household incomes tend to prioritize price over factors such as nutritional value when making food choices, compared to those with higher incomes [20]. Therefore, the presence of economic constraints suggests that individuals might be hindered from maintaining nutritionally balanced meals.

Regarding paying attention to nutritional balance when making food choices, univariate analysis showed significant associations between PC1 and PC3. However, multivariate analysis did not reveal any significant associations. In the simple regression model, a positive association was observed with PC1, while a negative association was observed with PC3. However, for PC2, a positive association was indicated in the simple regression model, but it turned into a negative association in the multivariate analysis. One possible interpretation is the effect of adjusting for demographic characteristics. The demographic characteristics of PC2 included younger age and male sex. Additionally, individuals with higher BMI scores were more prevalent in this group. Females generally tend to make healthier food choices than men and are more concerned about healthy eating behaviors to maintain their physique [39]. Since those who had higher PC2 scores had a predominance of younger males and higher BMI scores, they may have been less concerned with nutritional balance.

Taste was positively associated with both PC1 and PC3. It is crucial in food selection, with innate preferences for basic tastes such as sweet and bitter. These tastes are present in both healthy and unhealthy foods. Although taste often guides food choices, its link to specific dietary patterns remains unclear. Research suggests that prioritizing taste over health can lead to unhealthy food choices [40]. PC1 individuals prioritized taste, aimed for a balanced meal, and consumed it more frequently. Belief about the taste of unhealthy foods affects responses to health messages [41]. Taste is a key factor in food choice across cultures. Therefore, promoting healthy eating should focus on taste rather than health messages.

Food literacy encompasses the knowledge, attitudes, and skills required for informed dietary decisions and health impacts [42], significantly enhancing overall well-being [43]. Vidgen and Gallegos defined food literacy as a collection of interrelated knowledge, skills, and behaviors required to plan, manage, select, prepare, and eat food to meet needs and determine intake [43]. Multivariate regression analysis confirmed positive associations of attitudes and skills with PC1 and PC2 but a negative association between attitudes and PC3. College students with better meal preparation skills prepared more meals, including staples, main dishes, and side dishes [21]. Cooking for oneself is correlated with higher diet quality, whereas consuming commercial meals is associated with poorer quality [9]. Pregnant females with meal knowledge also had greater confidence in their meal preparation skills and were more likely to eat meals with staples, main dishes, and side dishes every day [27]. Although knowledge was associated with PC3, it may also be related to age. However, as food literacy suggests, knowledge alone does not guarantee behavioral change. PC1’s adoption of a favorable diet may stem from understanding balanced meals and maintaining positive attitudes. Therefore, fostering improved attitudes, skills, and confidence is crucial for behavioral changes.

Participants characterized by the PC3 pattern were less likely to consume a balanced meal consisting of staples, main dishes, and side dishes and exhibited lower SDQOL scores. A study involving adults aged 20 years and older found that those who adhered to a balanced meal reported a better subjective health status, regardless of demographic characteristics, time availability, or financial resources [29]. In our study, PC3 was negatively associated with SDQOL, even after adjusting for demographic variables and other factors. Conversely, PC1 exhibited a positive association, indicating a high frequency of balanced meal consumption. While causal relationships cannot be inferred due to the cross-sectional nature of this study, the findings suggest that individuals who felt burdened by adhering to a balanced meal did not practice such dietary habits, resulting in lower SDQOL scores.

### 4.2. Limitations

This study had some limitations that should be considered when interpreting the results. First, it was a secondary analysis of a cross-sectional survey of 2000 males and females aged 18–39 living nationwide, which was not conducted for the purpose of this study. Therefore, the items measuring food literacy, such as food knowledge and attitude, are limited and may be lacking. For example, meal preparation behaviors such as the frequency of cooking and preference for cooking have also been reported to be associated with meals that include staples, main dishes, and side dishes [22,27]. The ability to prepare good-tasting meals is an essential life skill and a major aspect of food literacy [43]. In addition, the frequency of eating out and using prepared meals have been reported to be associated with the practice of balanced meals [9,30]. Therefore, further studies are warranted. Second, the participants were recruited from a panel of an internet research company, and the survey was conducted online; web-based questionnaires with volunteer panels may feature recruitment and response biases [44]. However, the sample size was allocated by sex, age group, and region of residence according to the census results; students and individuals who had received professional education in nutrition or culinary arts were excluded from the study population. Respondents’ answers are subject to response bias. This study used the Likert method to assess participants’ views on eating a balanced meal, which may be affected by midpoint and extreme responses, as well as social desirability bias. However, to control for these biases, strategies such as avoiding ambiguous questions, mixing negative and positive items, and ensuring anonymity are recommended [45,46]. While this study incorporated several of these measures, it did not address noncontingent respondents who may not read the questionnaire carefully. Although trap questions can detect and exclude such respondents, they were not used in this web survey. Future research should implement these methods to improve data reliability and validity. Despite these limitations, this is the first study to measure the core components and patterns of values and related factors among young Japanese individuals toward eating a balanced meal to achieve the goal of a national health promotion program. This study revealed the need to consider socioeconomic conditions and other factors, emphasizing the importance of improving the eating environment, especially for those unwilling to actively adopt a balanced meal or who have a negative impression of such a diet, to achieve national health promotion goals.

Future studies should explore the impact of external factors, such as socioeconomic conditions and global events, such as the COVID-19 pandemic, on dietary behaviors. Additionally, longitudinal and intervention studies are recommended to examine changes in dietary values over time and to test strategies for promoting balanced meals. These approaches will provide a more comprehensive understanding of the factors influencing dietary behaviors.

### 4.3. Implications for Future Practices

This study revealed three core patterns in valuing the consumption of a complete set of staples, main dishes, and side dishes. It is essential to adopt an approach that matches the value patterns of the target population to promote desirable eating behaviors among young people. Individuals with higher PC1 scores tended to have a more balanced diet owing to their knowledge, skills, and attitudes toward meal planning. However, they also felt burdened. Therefore, nutrition professionals should provide recipes and skills training to encourage positive attitudes and improve meal preparation skills. Additionally, considering previous research findings [47] on convenience cooking products, such as meal bases and ready-made sauces, incorporating these into dietary interventions could offer time-saving solutions and alleviate the burden felt by individuals with higher PC1 scores. These products often include vegetable-rich recipes, addressing barriers of cooking skills, confidence, and creativity. Encouraging their use alongside skills training could be a practical approach to promoting healthier dietary habits among individuals with varying cooking skills and knowledge. Alternatively, for PC2, which was not associated with the intake of a balanced meal, and PC3, which was negatively associated, the following approach was considered: First, PC2, consisting mostly of young, single men living alone, was willing to use meals if available. However, they were not inclined to make food choices based on nutritional concerns. Therefore, strategies such as making vegetable dishes default side dishes at restaurants and delicatessens were considered effective. A previous study reported that creating a default healthy menu increased the selection rate of healthy menu items [48].

Given that PC3, who had a strong sense of burden related to consuming a combination of staple, main, and side dishes, also had limited financial resources, a strategy that leverages incentives, such as discounting set menus, may be a viable option. An intervention study that offered JPY 50 cashback to customers who ordered dishes with more vegetables reported an increase in their usage, especially among those with limited food budgets [49]. Although monetary incentives are relatively modest, this study indicates that incentives may be beneficial in reducing disparities in healthy food choices. Therefore, it is proposed that educational approaches and environmental improvements, such as modifying defaults and using economic incentives, are crucial. Policymakers, nutrition educators, and public health officials can implement these findings in real-world settings to improve dietary behaviors among young adults. Targeted interventions based on identified value patterns will vary depending on the situation and context of the target population, but considering target-specific strategies can help create an environment that supports healthier food choices.

## 5. Conclusions

This study identified three patterns of values among the younger generation regarding the consumption of a balanced meal consisting of staples, main dishes, and side dishes more than twice a day, as well as the core components of each pattern. In addition, the results revealed that sociodemographic factors, as well as nutritional knowledge, attitudes, skills, behaviors, and SDQOL, were related to each pattern. It is vital to adopt an approach that matches the value patterns of the target population to promote desirable eating behaviors among young people. Therefore, these findings could be a valuable resource for promoting nutrition education among younger generations. This study underscores the significance of reinforcing the values associated with practicing balanced meals to enhance their adoption among young Japanese adults. Furthermore, the use of defaults and incentives may be a valuable strategy for engaging a population less inclined to adhere to balanced meals. For future practice, such an integrated approach could serve as a pivotal instrument in promoting healthier dietary practices, thereby improving diet-related quality of life.

## Figures and Tables

**Table 1:** Characteristics of participants.

		*n*	%
Sociodemographic and anthropometric variables			
Age		29.7 ± 6.2	
Sex	male	960	50.8
	female	928	49.2
Living status	living alone	516	27.3
	living with others	1372	72.7
Marital status	single	1230	65.1
	married	658	34.9
Living with children	no	1417	75.1
	yes	471	24.9
Occupation	full-time employee/civil servant	999	52.9
	part-time job	350	18.5
	freelance/self-employed	71	3.8
	housewives/unemployed	217	11.5
	others (not specified)	251	13.3
Education	junior or senior high school	732	38.8
	junior college or vocational school	342	18.1
	college, university, graduate school	814	43.1
Household economic status	Financially constrained, causing significant distress	303	16.0
	Financial constrained, accompanied by some concern	621	32.9
	Financially constrained, yet little worrisome	781	41.4
	Financially secure, living worry-free	183	9.7
Body Mass Index (kg/m^2^)		21.5 ± 3.5	
BMI categories	underweight (BMI < 18.5)	365	19.3
	normal (BMI 18.5–24.9)	1266	67.1
	obese (25.0 ≤ BMI)	257	13.6

Mean ± standard deviation.

**Table 2:** Dietary knowledge, attitudes, skills, and behaviors and diet-related quality of life.

			*n*	%
Knowledge	Regarding eating a combination of staples, main dishes and side dishes			
		I do not know the term or its meaning	289	15.3
		I do not know the meaning, but I have heard the term	724	38.3
		I know both the term and its meaning	875	46.3
Attitude	Toward practicing healthy eating habits			
		not at all	199	10.5
		not so much	794	42.1
		sometimes	753	39.9
		always	142	7.5
	Toward eating a combination of staples, main dishes and side dishes on daily basis (1: not applicable at all, to 7: very applicable)		4.1 ± 1.5	

Motivations for food choices (1: not applicable at all, to 7: very applicable)				
		nutritional balance	4.2 ± 1.6	
		energy (calories)	4.0 ± 1.5	
		taste	4.9 ± 1.5	
		price	4.8 ± 1.5	
		seasonality	4.0 ± 1.5	
		safety	4.4 ± 1.5	
		appearance	4.1 ± 1.5	
		word-of-mouth	3.5 ± 1.6	
		popularity	3.4 ± 1.6	
		good manufacturer or store brand image	3.5 ± 1.5	
Skills (1: not applicable at all, to 7: very applicable)				
		considering the balance of nutrition and taste when planning a menu	3.8 ± 1.6	
		knowing what ingredients and utensils are needed for preparing meals	4.1 ± 1.6	
		reading nutrition labels and making food selections	3.8 ± 1.6	
		ability to prepare most dishes	4.0 ± 1.8	
		ability to prepare meals in a short time, even when busy	3.8 ± 1.6	
Behaviors	Eating a combination of meals at least twice a day			
		almost never	497	26.3
		two to three days a week	494	26.2
		four to five days a week	428	22.7
		almost everyday	469	24.8
Diet-related QOL	SDQOL (4~20 points)		13.7 ± 3.5	

Mean ± standard deviation. QOL: quality of life, SDQOL: subjective diet-related quality of life.

**Table 3:** Factor loadings of the 12 components regarding participants’ perceived values of eating a combination of staples, main dishes, and side dishes through principal component analysis with varimax rotation.

	PC1	PC2	PC3
I think it is good for our health.	**0.878**	0.090	0.285
I think that it improves nutritional balance.	**0.877**	0.068	0.299
It helps to feel that I am living a fulfilling life.	**0.828**	0.241	0.127
If such meal is prepared, I would like to proactively adopt it.	**0.733**	0.301	0.289
It helps to facilitate communication with family members.	**0.572**	**0.488**	−0.182
If there are places where such meals can be purchased or eaten near my home or commute route, I would like to incorporate them.	0.109	**0.872**	0.185
If there is a service that delivers such meals at a reasonable price, I would be willing to adopt it.	0.059	**0.819**	0.243
If such meals are offered at a lower price at workplace, I would be willing to select it.	0.183	**0.803**	0.259
If I have someone to eat with, I’m willing to eat such meals.	**0.429**	**0.662**	−0.138
I feel that preparing such meals is a waste of time.	0.040	0.265	**0.791**
I feel that preparing such meals is troublesome.	**0.483**	0.088	**0.757**
I think that adopting such meals cost a lot money.	**0.544**	0.107	**0.687**
Eigenvalue	5.85	1.90	1.26
Explained variance (%)	48.73	15.85	10.52
Cumulative variance explained (%)	48.73	64.58	75.10

Kaiser–Meyer–Olkin (KMO): 0.879. PC1: Valuable yet burdensome, PC2: Environment-reliant, weak initiative, and PC3: Low value due to hassle. Factor loadings exceeding +0.40 or falling below −0.40 are highlighted in bold.

**Table 4:** Linear regression coefficients (β) from simple and multiple analyses examining perception patterns in relation to participants’ sociodemographics and anthropometric variables, knowledge, attitudes, skills, behaviors, and diet-related QOL among young Japanese adults.

		PC1: Valuable Yet Burdensome		PC2: Environment-Reliant, Weak Initiative		PC3: Low Value due to Hassle	
		Simple	Multiple	Simple	Multiple	Simple	Multiple
Sociodemographic and anthropometric variables							
	Age	0.053 *	-	−0.052 *	−0.060 **	0.009	0.052 *
	Sex (1: Male, 2: Female)	0.150 ***	0.040 *	−0.062 **	−0.070 ***	−0.018	-
	Living status (1: living alone, 2: living with others)	0.118 ***	-	−0.036	−0.077 ***	−0.089 ***	-
	Marital status (1: single, 2: married)	0.149 ***	0.052 **	0.043	0.056 *	−0.121 ***	−0.083 ***
	Living with children (1: no, 2: yes)	0.105 ***	-	0.027	-	−0.062 **	-
	Occupation 1 (1: full-time employee/civil servant, 0: all other)	−0.048 *	-	0.084 ***	-	−0.020	-
	Occupation 2 (1: part-time job, 0: all other)	0.016	-	−0.034	-	0.009	-
	Occupation 3 (1: freelance/self-employed, 0: all other)	−0.009	-	0.011	-	0.016	-
	Occupation 4 (1: housewives/unemployed, 0: all other)	0.134 ***	-	−0.013	-	−0.052 *	-
	Education (1: junior or senior high school, 2: junior college or vocational school, 3: college, university or graduate school)	0.030	-	0.040	-	−0.076 ***	-
	Household economic status (1: Financially constrained, causing significant distress~4: Financially secure, living worry-free)	0.045	-	0.044	-	−0.160 ***	−0.065 **
	Body Mass Index	−0.047 *	-	0.045	0.048 *	0.006	-
Knowledge (1: I don’t know the term or its meaning, to 3: I know both the term and its meaning)							
	Regarding eating a combination of staple food, main dishes, and side dishes	0.297 ***	0.091 ***	0.050 *	-	0.045	0.095 ***
Attitudes							
	Toward practicing healthy eating habits (1: not at all, to 4: always)	0.197 ***	-	0.105 ***	-	−0.132 ***	−0.054 *
	Toward eating a combination of staple food, main dish and side dishes on daily basis (1: not applicable at all, to 7: very applicable)	0.360 ***	0.090 ***	0.294 ***	0.105 ***	−0.175 ***	−0.142 ***
Motivations for food choices (1: not applicable at all, to 7: very applicable)							
	nutritional balance	0.363 ***	-	0.255 ***	−0.116 **	−0.098 ***	-
	energy (calories)	0.248 ***	-	0.314 ***	0.075 *	−0.062 **	-
	taste	0.538 ***	0.259 ***	0.088 ***	−0.056 *	0.110 ***	0.103 ***
	price	0.479 ***	0.199 ***	0.091 ***	-	0.228 ***	0.298 ***
	seasonality	0.322 ***	0.053 *	0.344 ***	0.104 ***	−0.141 ***	−0.168 ***
	safety	0.383 ***	-	0.238 ***	-	−0.025	-
	appearance	0.316 ***	-	0.295 ***	-	−0.059 *	-
	word-of-mouth	0.074 **	−0.079 **	0.440 ***	-	−0.071 **	-
	popularity	0.025	−0.110 ***	0.463 ***	0.142 ***	−0.079 ***	-
	good manufacturer or store brand image	0.058 *	-	0.408 ***	0.240 ***	−0.066 **	0.061 *
Skills (1: not applicable at all, to 7: very applicable)							
	considering the balance of nutrition and taste when planning a menu	0.287 ***	-	0.274 ***	-	−0.117 ***	-
	knowing what ingredients and utensils are needed for preparing meals	0.365 ***	0.114 ***	0.190 ***	-	−0.035	-
	reading nutrition labels and making food selections	0.229 ***	-	0.304 ***	0.097 ***	−0.056 *	-
	ability to prepare most dishes	0.313 ***	-	0.219 ***	-	−0.058 *	-
	ability to prepare meals in a short time, even when busy	0.263 ***	-	0.262 ***	-	−0.096 ***	-
Behaviors (1: almost never, to 4: almost every day)							
	Eating a combination of staple foods, main dishes, and side dishes at least twice a day	0.212 ***	0.043 *	0.067 **	-	−0.203 ***	−0.122 ***
Diet-related QOL							
	SDQOL (4~20 points)	0.343 ***	0.138 ***	0.092 ***	-	−0.144 ***	−0.129 ***
	Adjusted R^2^		0.411		0.268		0.195

* *p* < 0.05, ** *p* < 0.01, *** *p* < 0.001. Stepwise model. standardized regression coefficient (β). QOL: quality of life, SDQOL: subjective diet-related quality of life.

## Data Availability

Data are contained within the article.
